# A novel tetrahydrocurcumin derivative as a potential anti-colorectal cancer agent: studies on its design, synthesis, and in vitro and in vivo biological evaluation

**DOI:** 10.1007/s13659-026-00639-7

**Published:** 2026-08-10

**Authors:** Yixin Chen, Ao Duan, Wenzhang Li, Meitao Duan, Jiahui Yu, Ahmed Mahal, Yongyan Zhu, Quanhong Zhu

**Affiliations:** 1 School of Traditional Chinese Medicine, Southern Medical University, Guangzhou, 510515 China; 2 Guangdong Provincial Key Laboratory of Chinese Medicine Pharmaceutics, Guangzhou, 510515 China; 3 School of Pharmacy, Xiamen Medical College, Xiamen, 361023 China; 4 Department of Medical Biochemical Analysis, College of Health Technology, Cihan University Erbil, Erbil, Kurdistan Region Iraq

**Keywords:** THC derivatives, α, β-unsaturated carbonyl, CRC, PI3K/AKT/mTOR signaling pathway, Apoptosis

## Abstract

Objective: To design and synthesize a series of tetrahydrocurcumin (THC) derivatives bearing α, β-unsaturated carbonyl moieties, evaluate their anticancer activities, screen potential candidates for colorectal cancer (CRC) therapy, and assess their biological activities. Methods: THC derivatives were synthesized in high yield via Knoevenagel condensation reaction, and their anticancer activities were evaluated in HCT116, SW480, SW620, HeLa, A549 and HepG2 cell lines. The in vitro and in vivo anti-CRC activities of the target compound were subsequently investigated. Results: Most compounds displayed potent antiproliferative activity against multiple tumor cell lines, with notably enhanced sensitivity in CRC cells. The fluorinated derivative **5 (C5)** exhibited superior anti-CRC activity (IC_50_ = 2.66 ± 0.82 μM) relative to the positive control 5-fluorouracil (5-FU). By contrast, its reduced product compound **H5** showed a 13-fold decline in potency (IC_50_ = 35.74 ± 1.40 μM), validating that the α, β-unsaturated carbonyl moiety represents the critical pharmacophore. Furthermore, **C5** induced S-phase cell cycle arrest in HCT116 cells, triggered apoptosis via modulation of Bcl-2 family proteins, and suppressed cell migration. Mechanistically, it exerted its anticancer effects by inhibiting the PI3K/AKT/mTOR signaling pathway through downregulating p-AKT and p-mTOR. In vivo, **C5** effectively inhibited tumor growth and cancer cell proliferation in HCT116 xenograft models, with higher efficacy and lower toxicity than 5-FU. Conclusions: These THC derivatives exhibited selective targeting toward CRC cells and suppressed the PI3K/AKT/mTOR signaling pathway, exerting significant anti-CRC efficacy both in vitro and in vivo, and thus held promise as potential lead compounds for CRC therapy.

**Graphical abstract:**

**Figure:**
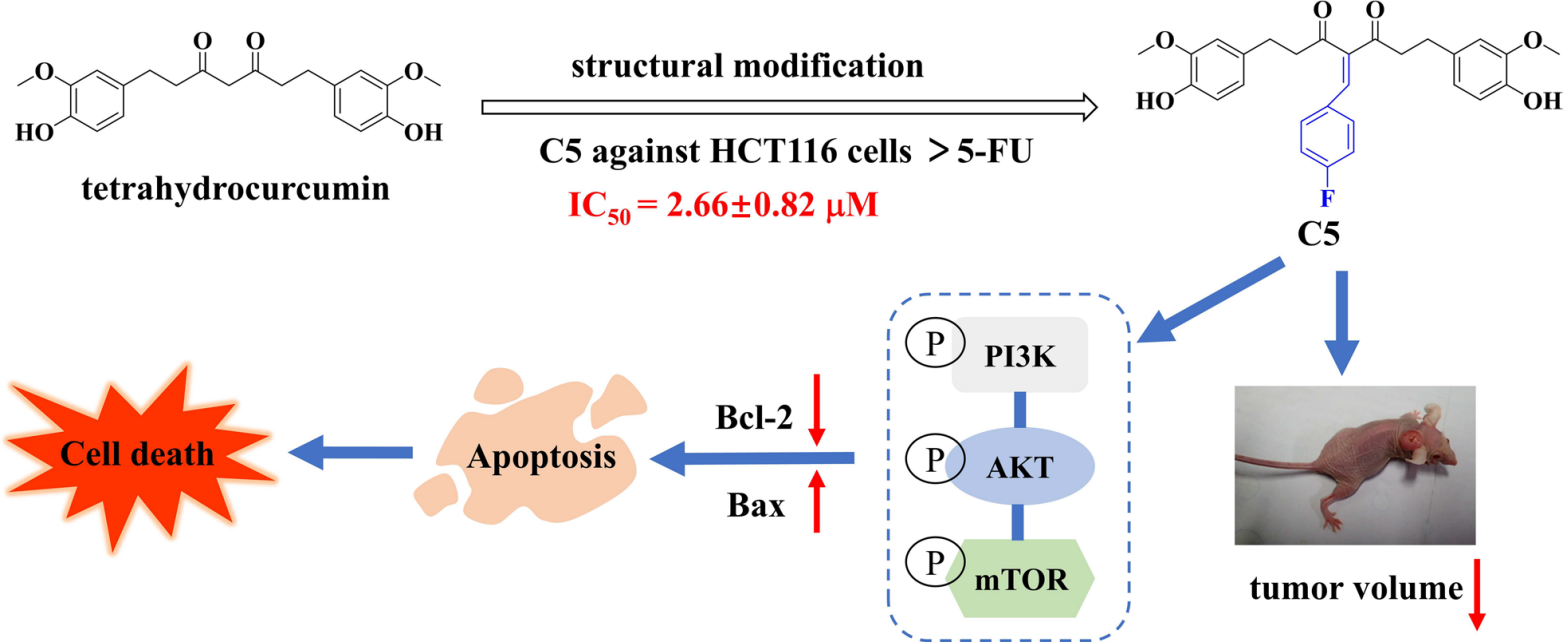

**Supplementary Information:**

The online version contains supplementary material available at 10.1007/s13659-026-00639-7.

## Introduction

Colorectal cancer (CRC) is a malignant tumor of the digestive tract that occurs in the colorectum, posing a serious threat to human health. According to the 2022 global cancer statistics, CRC ranks third in incidence and second in mortality among all malignant tumors worldwide [1]. It is projected that by 2030, the number of CRC cases worldwide will increase by 60%, reaching over 2.2 million new cases and 1.1 million cancer-associated deaths [2]. This demonstrates the severe challenges in CRC diagnosis and treatment, with significant impacts on human life and societal development. Currently, conventional treatments for CRC include surgery, chemotherapy, radiotherapy, immunotherapy. Although multiple therapeutic options exist, chemoresistance and the toxic side effects of CRC chemotherapy cause substantial harm to patients, limiting their clinical application. Therefore, developing more drugs that can efficiently kill cancer cells while remaining safe and minimally toxic has become a critical issue in current anti-CRC drug research.

Finding lead compounds from traditional Chinese medicines, natural products, and their bioactive metabolites is an effective strategy for drug discovery. Curcumin (CUR) is the primary active component derived from traditional Chinese medicines such as *Curcuma longa* L. and *Curcuma aromatica* Salisb., and its potent anticancer effects have been well documented [3, 4]. However, poor systemic bioavailability resulting from chemical instability, low absorption, rapid metabolism, and fast elimination limits its druggability as a drug candidate [5]. Tetrahydrocurcumin (THC), an active metabolite of curcumin in vivo [6], exhibited superior chemical stability, bioavailability and in vivo antitumor effects compared with curcumin [7–9]. Studies have demonstrated that THC reduced the number of aberrant crypt foci (ACF) in azoxymethane (AOM)-induced mouse colorectal cancer models, and was more effective than curcumin for inhibiting colorectal tumor development [10]. THC exerted a more potent anti-angiogenic and anti-metastatic effects on HepG2 hepatocellular carcinoma and CaSki cervical cancer xenografts in BALB/c nude mice compared with curcumin [11, 12]. Moreover, THC could suppress the growth of gliomas in xenograft mouse models by reducing glutathione (GSH) levels, thereby exerting anti-proliferative effects on glioma cells [13]. Intravenous administration of tetrahydrocurcumin in an osteosarcoma-induced pulmonary metastasis model in nude mice significantly reduced the number of metastatic nodules, demonstrating its anti-metastatic properties [14]. These findings suggests that tetrahydrocurcumin has superior in vivo biological effects compared with curcumin, possibly due to its ability to regulatory effects on tumor cell cycle arrest, apoptosis, and autophagy [15–17].

In recent years, the introduction of Michael acceptors in structural modification of natural products has been shown to be an effective strategy for optimizing and improving biological activity. The α, β-unsaturated carbonyls are common Michael acceptors that react with various thiols in cellular proteins, leading to a series of biological activities [18]. The A-ring modified 18β-glycyrrhetinic acid derivative featuring an α, β-unsaturated carbonyl group demonstrated potent antiproliferative activity against human colorectal carcinoma HCT116 cells (IC_50_ = 0.14 ± 0.02 μM) [19]. An α, β-unsaturated carbonyl group was introduced into ring A of andrographolide while retaining the original Michael acceptor α-alkylidene-γ-butyrolactone moiety, significantly enhancing growth inhibition against HCT116 cells (IC_50_ = 5.50 ± 0.14 μM) [20]. Introducing electrophilic Michael acceptors into oleanolic acid also showed increasing reactivity towards cellular targets, thereby enhancing antitumor [21] and anti-inflammatory activities [22], for example, 2-cyano-3,12-dioxooleana-1,9(11)-dien-28-oic acid (CDDO) and its methyl ester CDDO-Me have been shown to possess effective anticancer activity (IC_50_ = 0.26 ± 0.02 μM for HepG2 cells). CDDO-Me is currently in phase III clinical trials for the treatment of chronic kidney disease in patients with type II diabetes [23]. In addition, constructing α, β-unsaturated carbonyl group in the structure of curcumin could also improve the antitumor activity, especially against human lung carcinoma QG-56 cells (IC_50_ = 12.5 μM) [24].

This study aims to structurally modify THC by introducing α, β-unsaturated carbonyl groups to enhance its anticancer activity through increased protein interactions. We will also investigate the selectivity and structure–activity relationships of the synthesized compounds against different cancer cell lines, and evaluate their in vitro and in vivo anticancer effects, as well as the potential mechanisms of these derivatives.

## Results and discussion

### Chemistry

**Scheme 1:**
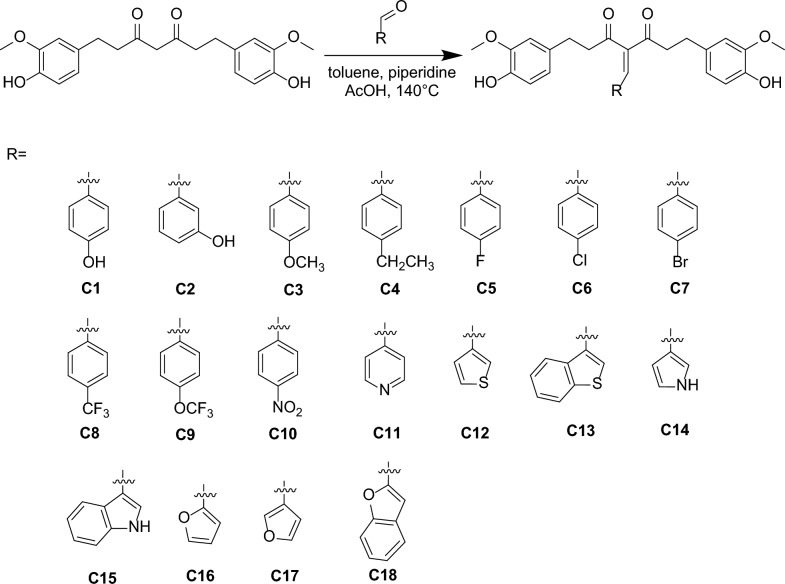
Synthesis of the target compounds **C1-C18**

**Scheme 2:**
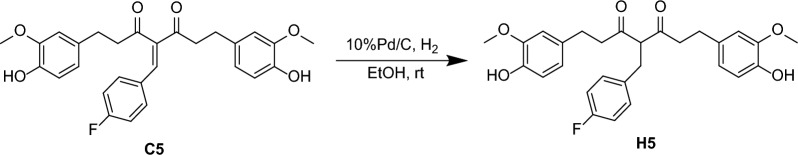
Synthesis of compound **H5**

As depicted in Scheme 1, the Knoevenagel condensation reaction was employed. Using piperidine and acetic acid as catalysts, the active methylene group in THC reacted with various substituted aromatic aldehydes, leading to the dehydration and formation of THC derivatives **C1–C18** bearing α, β-unsaturated carbonyl groups. Taking compound **C5** as an example, its hydrogenation yielded in compound **H5** (Scheme 2). The structures of the target compounds were characterized by ^1^H NMR, ^13^C NMR, and HRMS.

### Biological activity

#### Antiproliferative activity in vitro

The in vitro antiproliferative activities of compounds **C1–C18** against four human tumor cell lines (HCT116, HeLa, A549, and HepG2) were evaluated using the MTT assay. Cisplatin was used as a positive control, and the IC_50_ values are listed in Table 1.

**Table 1:** IC_50_^a^ of tetrahydrocurcumin derivatives C1-C18 against different cell lines for 72 h

Compd.	Cancer cell lines IC_50_ ± SD (μM)						Non-cancer cell lines IC_50_ ± SD (μM)
	Hela	A549	HepG2	HCT116	SW480	SW620	NCM460
THC	29.75 ± 0.39	73.05 ± 4.47	52.74 ± 0.27	53.03 ± 0.78	54.28 ± 1.50	77.41 ± 2.51	52.09 ± 0.43
C1	6.76 ± 0.11	12.07 ± 0.13	22.00 ± 1.90	4.26 ± 0.10*	2.61 ± 0.02	5.08 ± 0.04	11.35 ± 0.27
C2	6.80 ± 0.14	11.94 ± 0.40	21.21 ± 0.21	2.61 ± 1.50***	2.79 ± 1.44	1.95 ± 0.13	9.64 ± 0.38
C3	8.93 ± 0.15	11.73 ± 0.2	20.61 ± 1.00	4.27 ± 2.20*	4.59 ± 0.40	4.31 ± 0.20	23.35 ± 1.27
C4	17.19 ± 0.19	23.83 ± 0.51	33.24 ± 0.80	10.68 ± 0.10	7.14 ± 0.20	6.53 ± 0.24	50.80 ± 2.95
C5	9.07 ± 0.27	52.02 ± 6.24	33.52 ± 2.21	2.66 ± 0.82***	3.16 ± 0.20	3.98 ± 0.11	26.85 ± 0.55
C6	5.96 ± 0.46	8.19 ± 2.46	23.06 ± 0.24	2.49 ± 1.23***	3.43 ± 1.21	3.31 ± 0.09	16.53 ± 0.16
C7	10.56 ± 0.60	28.46 ± 1.99	22.04 ± 0.64	3.48 ± 1.16**	5.66 ± 0.17	6.03 ± 0.10	28.69 ± 0.43
C8	21.06 ± 0.10	32.73 ± 0.82	58.39 ± 3.88	6.47 ± 0.83	5.07 ± 0.50	13.58 ± 0.33	42.18 ± 1.53
C9	31.33 ± 1.03	32.87 ± 0.21	42.04 ± 1.85	15.27 ± 0.60	10.51 ± 0.29	16.12 ± 0.71	43.23 ± 0.19
C10	7.63 ± 0.23	12.33 ± 0.33	26.78 ± 0.17	5.80 ± 1.57	5.11 ± 0.28	4.66 ± 0.42	9.45 ± 0.60
C11	8.78 ± 0.12	24.65 ± 0.41	29.12 ± 1.45	8.73 ± 0.30	2.90 ± 0.19	4.13 ± 0.33	14.76 ± 0.24
C12	8.91 ± 0.31	37.36 ± 4.02	26.58 ± 2.42	4.97 ± 1.05	4.25 ± 0.10	8.59 ± 0.12	21.45 ± 0.37
C13	5.75 ± 0.11	10.16 ± 0.79	28.08 ± 0.12	2.76 ± 0.54***	2.98 ± 0.69	5.33 ± 0.50	17.57 ± 0.10
C14	59.76 ± 0.60	56.18 ± 1.35	86.85 ± 6.74	16.12 ± 0.18	49.37 ± 0.92	54.83 ± 4.19	46.67 ± 0.62
C15	19.91 ± 0.19	14.78 ± 0.12	41.77 ± 1.52	10.70 ± 0.80	14.38 ± 0.32	31.19 ± 0.20	29.38 ± 2.08
C16	25.81 ± 1.03	37.01 ± 0.20	38.92 ± 2.88	26.22 ± 1.19	33.30 ± 1.20	34.07 ± 0.14	46.96 ± 1.04
C17	16.22 ± 0.23	24.01 ± 0.14	30.29 ± 0.71	21.64 ± 1.37	8.89 ± 0.21	12.98 ± 0.23	34.65 ± 0.32
C18	7.63 ± 0.10	26.83 ± 0.50	35.53 ± 0.47	5.19 ± 1.38	4.78 ± 0.13	8.77 ± 0.38	25.07 ± 0.14
H5	NT	NT	NT	35.74 ± 1.40^###^	NT	NT	NT
5-FU	NT^b^	NT	NT	4.23 ± 0.33	5.32 ± 0.71	2.73 ± 0.17	20.43 ± 1.83
Cisplatin	6.82 ± 0.34	16.22 ± 0.67	12.56 ± 0.82	9.32 ± 0.93	8.90 ± 0.54	10.02 ± 0.20	> 200

^a^IC_50_: The concentration that causes 50% inhibition of cell proliferation. Data are expressed as the means ± SD from triplicate determination from three independent experiments

^b^NT: Not test

^*^Compared with cisplatin, *p < 0.05, **p < 0.01, ***p < 0.001; ^#^compared with C**5**, ^###^*p* < 0.001

The results demonstrated that, with the exception of compound **C14**, almost all compounds exhibited stronger antiproliferative activities against the four cancer cell lines compared with the parent compound THC. The antiproliferative activity was most potent against HCT116 cells, followed by HeLa, A549, and HepG2 cells. For the HCT116 cell line, the antiproliferative activities of all compounds were superior to those of THC. Compounds **C1**, **C2**, **C3**, **C5**, **C6**, **C7**, and **C13** displayed significantly better antiproliferative activities than the positive control cisplatin (*p* < 0.05), which bore hydroxyl, methoxy, and halogen electron-withdrawing substituents. This finding indicated that the introduction of electron-withdrawing groups significantly enhanced the antiproliferative activity of THC. Among these, the halogen-substituted compounds (**C5–C7**) exhibited particularly strong antiproliferative activity. This was especially prominent in **C5** (IC_50_ = 2.66 ± 0.82 µM), which was approximately 20 times more potent than THC (IC_50_ = 53.03 ± 0.78 µM) and about 5 times more potent than cisplatin (IC_50_ = 9.32 ± 0.93 µM).

We selected three colorectal cancer cell lines (HCT116, SW480, SW620) to test the in vitro antiproliferative activities of the compounds and to investigate whether they exhibited selectivity towards colorectal cancer cells. The clinically used first-line drug 5-FU was employed as a positive control, and the IC_50_ values were listed in Table 1.

The results indicated that after the introduction of α, β-unsaturated carbonyl group into the structure of THC, the compounds exhibited significant inhibitory activity on the proliferation of the three colorectal cancer cell lines, with antiproliferative activities superior to those of THC, approximately 1.4**–**40 times higher than THC, with IC_50_ values for THC inhibition of HCT116, SW480, and SW620 proliferation being 53.03 ± 0.78 µM, 54.28 ± 1.50 µM, and 77.41 ± 2.51 µM, respectively. Approximately 50–60% of the compounds had antiproliferative activities at least 10 times higher than THC, suggesting that these compounds possessed selectivity towards colorectal cancer cells. Among the three colorectal cancer cell lines, the antiproliferative activity of the compounds was most potent against HCT116 and SW480 cells, followed by SW620. The proportion of compounds with IC_50_ values below 10 µM were 67%, 78%, and 67%, respectively, and compound **C2** displayed stronger antiproliferative activity than 5-FU. In addition, the antiproliferative activities of compounds **C5**, **C6**, **C7**, and **C13** against HCT116 cells were superior to that of 5-FU (IC_50_ = 4.23 ± 0.33 µM), and the activities of compounds **C1**, **C5**, **C6**, **C11**, **C12**, **C13**, and **C18** against SW480 cells were superior to that of 5-FU (IC_50_ = 5.32 ± 0.71 µM).

Upon analysis of the structure–activity relationship, it has been observed that compounds with electron-withdrawing effects generally exhibited stronger antiproliferative activity compared to those with electron-donating effects. For instance, in the series of compounds containing a phenyl ring (**C1–C10**), those with electron-withdrawing groups at the para-position of the phenyl ring demonstrated superior activity to those with electron-donating groups. This was particularly evident for compounds containing hydroxyl or methoxy groups (**C1–C3**), as well as those containing halogen atoms (**C5–C7**), which displayed enhanced antiproliferative activity against colorectal cancer cells. In the aromatic heterocyclic systems, compounds with electron-donating effects, such as pyridine, benzo[b]thiophene, and thiophene (**C11–C13**), showed significantly better antiproliferative activity than electron-withdrawing five-membered heterocyclic compounds, such as pyrrole, furan, and indole (**C14–C17**). This could be attributed to the electron-withdrawing groups' ability to enhance the reactivity of the β-carbon atoms in α, β-unsaturated carbonyl groups, thereby increasing the activity of Michael addition reactions with nucleophilic groups in cellular proteins and DNA. Consequently, this results in the amplification of inhibitory activity against tumor cell proliferation.

To further demonstrated that the α, β-unsaturated carbonyl group was an effective pharmacophore for enhancing the antitumor activity of parent compounds, we reduced the double bond of the α, β-unsaturated carbonyl group through hydrogenation and compared the inhibitory activity against HCT116 cell proliferation. Taking compound **C5** as an example, the compound which exhibited significant inhibitory activity against three types of colorectal cancer cells, its hydrogenation resulted in compound **H5** (Scheme 2). Then, the antiproliferative activity of **H5** against HCT116 cells was evaluated. As shown in Table 1, compared to **C5** (IC_50_ = 2.66 ± 0.82 µM), **H5** exhibited a considerably higher IC_50_ value of 35.74 ± 1.40 µM against HCT116 cells (*p* < 0.05), approximately 13 times higher, with markedly weakened proliferative inhibitory activity. This trend was consistent with the patterns reported in previous literature [25], suggesting that the significantly decreased activity of **H5** mainly arises from the loss of its ability to undergo Michael addition with intracellular thiols. This further indicated that constructing α, β-unsaturated carbonyl group within the THC structure could effectively enhance its antitumor activity, and the α, β-unsaturated carbonyl group may be an essential pharmacophore for the significant antitumor activity of THC derivatives, which mainly exerts its effect through covalent interactions with thiol-containing proteins [26, 27].

**Fig. 1:**
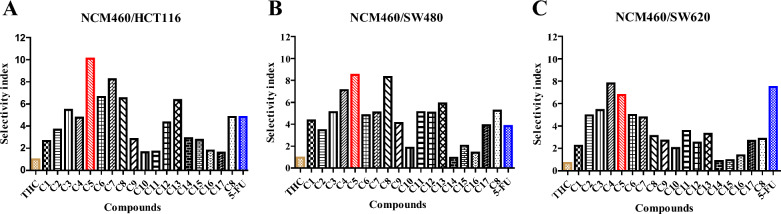
Selectivity index (SI) of NCM460 cells to HCT116, SW620 and SW480 cells. **A** SI = [IC_50_ (NCM460)]/[IC_50_ (HCT116)]; **B** SI = [IC_50_ (NCM460)]/[IC_50_ (SW480)]; **C** SI = [IC_50_ (NCM460)]/[IC_50_ (SW620)]

Moreover, we examined the toxicity of the compounds against normal cells using intestinal epithelial cells (NCM460) and studied the selectivity of the compounds against three types of colorectal cancer cells. The results depicted in Fig. 1 revealed that the selectivity index (SI) of THC for the three colorectal cancer cells was less than 1. With the exception of compound **C14**, the SI values of the structurally modified compounds were higher than that of THC and greater than 1, indicating that these compounds exhibited lower cytotoxicity against normal intestinal epithelial cells and possessed stronger antitumor activity with improved safety. Notably, the SI values of **C5** for HCT116 and SW620 cells were higher than those of the positive control 5-FU (SI = 4.83), and **C5** demonstrated the best selectivity for the HCT116 cell line (SI = 10.09), approximately twice that of 5-FU. This suggested that **C5** possessed stronger antiproliferative activity against colorectal cancer HCT116 cells and lower cytotoxicity against normal intestinal epithelial NCM460 cells. Consequently, among all the compounds, we selected **C5**, which exhibited the best antitumor activity and safety profile, for further research. **C5** is a compound with a fluorine atom at the para-position of the phenyl ring. Fluorine is one of the most frequently introduced functional groups in small molecule drug design and occupies a significant position in small molecule drugs, such as lapatinib and sofosbuvir, among other blockbuster medications. The proportion of fluorine-containing small molecule drugs approved by the FDA has increased from an average of 20% before 2010 to an average of 30% annually. Particularly in 2018, the proportion of fluorine-containing drugs that passed FDA approval rose to 43%.

#### C5 inhibited the clonal proliferation of HCT116 cells

**Fig. 2:**
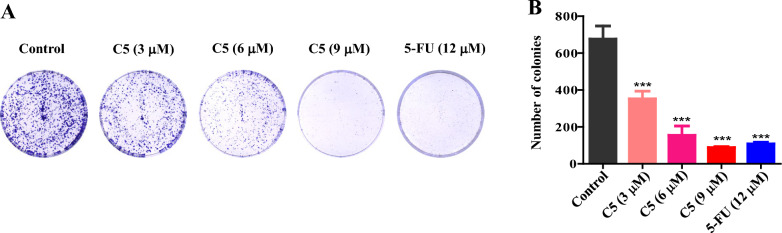
Effect of **C5** on clonal proliferation of HCT116 cells. **A** **C5** inhibited colorectaly formation of HCT116 cells; **B** Colorectaly number was measured (mean ± SD, n = 3), *Compared with the control group, *p < 0.05, **p < 0.01, ***p < 0.001

As shown in Fig. 2, the results of the colony formation assay showed that HCT116 cells in the blank control group were dense, with a cell colony count of 676.00 ± 40.95. After incubation with **C5** for 48 h, the number of colonies significantly decreased with increasing drug concentrations. At a concentration of 9 µM, the colony formation of HCT116 cells was almost completely inhibited. The cell colony counts for **C5** at concentrations of 3, 6, and 9 µM were 352.70 ± 23.78, 155.33 ± 28.59, and 88.22 ± 2.85, respectively, exhibiting significant differences compared with the blank control group (*p* < 0.05). The effect of **C5** on colony formation at a concentration of 9 µM was superior to that of the positive control 5-FU at a concentration of 12 µM (108.30 ± 5.21).

#### C5 induced S phase arrest in HCT116 cells

**Fig. 3:**
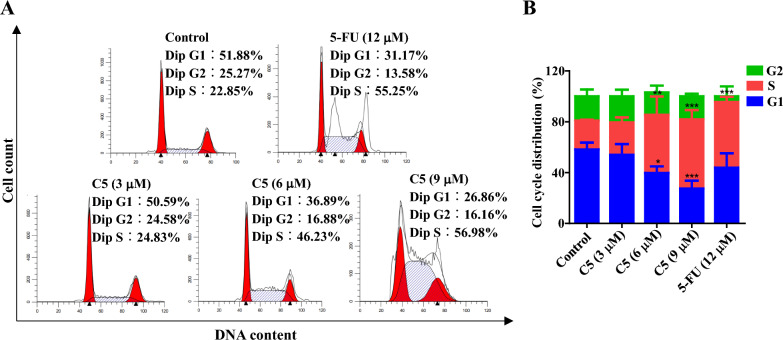
Effect of **C5** on the cell-cycle distribution in HCT116 cells. **A** Cells were treated with 0.1%DMSO (Control)、5-FU (12 μM) or **C5** (3, 6, and 9 μM) for 48 h, collected, stained with PI (propidium iodide)and then analyzed by flow cytometry; **B** The percentages of cells in G0/G1, S and G2/M phases were determined (mean ± SD, n = 3), *Compared with the control group, *p < 0.05, **p < 0.01, ***p < 0.001

Using 5-FU as a positive control, the effect of **C5** on the cell cycle of HCT116 cells was analyzed using flow cytometry. As shown in Fig. 3, the percentage of S phase cells in the blank control group was 22.85%. After treatment with** C5** at concentrations of 3, 6, and 9 µM for 48 h, the percentage of S phase cells increased to 24.83%, 46.23%, and 56.98%, respectively, displaying a dose-dependent accumulation. The S phase arrest effect in the high concentration group (9 µM) was comparable to that of 12 µM 5-FU (55.25%). These results suggested that **C5** might cause damage to the DNA of HCT116 cells, inducing cell cycle arrest at the S phase. The strength of this effect was positively correlated with the concentration of **C5**.

#### C5 induced apoptosis in HCT116 cells

Apoptosis plays a crucial role in the development and formation of tumors. To determine whether **C5** inhibited cell growth by inducing apoptosis, we used the Annexin V-FITC and PI double staining method and analyzed the percentage of apoptotic HCT116 cells by flow cytometry.

**Fig. 4:**
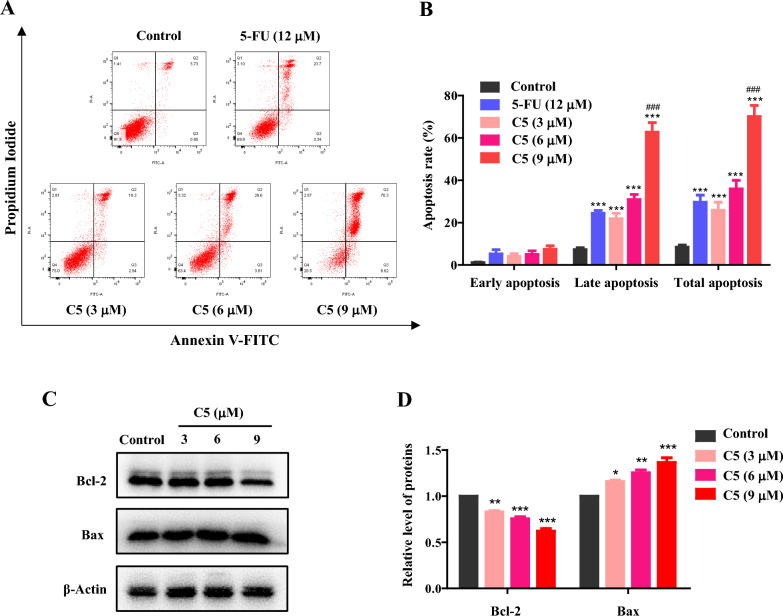
Effect of **C5** on HCT116 cells apoptosis in vitro. **A** HCT116 cells were incubated with 0.1%DMSO (control)、5-FU (12 μM) or **C5** (3, 6,  and 9 μM) for 48 h, harvested and stained with Annexin V and PI, then the percentages of apoptotic cells were determined by flow cytometry; **B** Apoptosis rate was displayed through the histogram (mean ± SD, n = 3). *Compared with the control group, *** *p* < 0.001; ^#^Compared with the 5-FU group, ^###^ *p* < 0.001; **C** Effect of **C5** on the expression of apoptotic proteins; **D** Relative levels of apoptosis-related proteins were determined (mean ± SD, n = 3), *Compared with the control group, * *p* < 0.05, ** *p* < 0.01, *** *p* < 0.001

As shown in Fig. 4A, B, the percentage of total apoptotic cells (including early and late apoptosis) in the control group of HCT116 cells was 6.68%. After treatment with **C5** at concentrations of 3, 6, and 9 µM for 48 h, the percentage of total apoptotic cells significantly increased to 22.14%, 33.21%, and 76.92%, respectively. The high-concentration **C5** group (9 µM) induced apoptosis significantly more effectively than the 12 µM 5-FU group (27.04%) (*p* < 0.001), while the medium-concentration **C5** group showed an apoptotic effect comparable to that of 5-FU. Notably, 5-FU exerted its predominant anti-tumor activity via cell cycle arrest rather than potent apoptosis induction [28]. Thus, even at the relatively high concentration of 12 μM, the pro-apoptotic activity of 5-FU is still weaker than that of high-concentration **C5.** In addition, we found that **C5** mainly induced late stage apoptosis in cells, with 85% of total apoptotic cells in the late stage. This suggested that **C5** might exert antitumor effect by inducing apoptosis, primarily occurring in the late stage and in a dose-dependent manner.

As key upstream regulatory proteins of the mitochondrial apoptotic pathway, the Bcl-2 family proteins play a critical role in apoptosis. A decrease in the Bcl-2/Bax ratio serves as compelling evidence for the activation of the mitochondrial apoptosis pathway and also marks an increased cellular sensitivity to apoptosis [29, 30]. Further observation of the effect of **C5** on the expression of anti-apoptotic protein Bcl-2 and pro-apoptotic protein Bax was conducted. Western blot results showed that, compared with the control group, after treatment with **C5**, the expression of anti-apoptotic protein Bcl-2 in HCT116 cells was significantly downregulated, while the expression of pro-apoptotic protein Bax was significantly upregulated in a dose-dependent manner (Fig. 4C, D). Studies have shown that compounds containing an α, β-unsaturated carbonyl group induced caspase-dependent endogenous apoptosis in non-small cell lung cancer cells by regulating the expression of Bcl-2 family proteins, mediating mitochondrial damage, and activating the Caspase-9/3 pathway [31, 32]. This suggested that **C5** induced apoptosis in HCT116 cells by regulating Bcl-2 family proteins, indicating that it may act through a caspase-dependent endogenous apoptotic pathway.

#### C5 inhibited migration of HCT116 cells

**Fig. 5:**
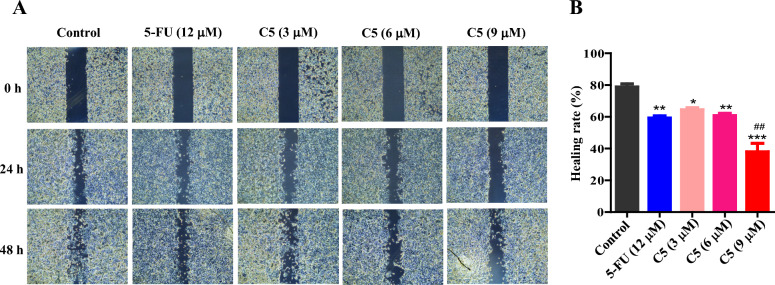
Effect of **C5** on migration of HCT116 cells. **A** Cell migration was detected by wound scratch assay. The representative images of cell migration in the wound scrape model at 0, 24, and 48 h (100 ×); **B** Statistical analysis of 48-h scratch test (mean ± SD, n = 3), *Compared with the control group, * *p* < 0.05, ** *p* < 0.01, *** *p* < 0.001; ^#^Compared with the 5-FU group, ^##^ *p* < 0.01

To investigate whether **C5** could inhibit tumor cell migration, a wound healing assay was employed to evaluate the inhibitory effect of **C5** on HCT116 cell migration using the healing rate as an indicator. As depicted in Fig. 5, the healing rate in the control group was 78.81 ± 1.99%. After treatment with **C5** at concentrations of 3, 6, and 9 µM, the wound healing extent of the cells was significantly decreased, with healing rates of 64.46 ± 1.22%, 60.76 ± 1.42%, and 37.94 ± 5.39%, respectively. Notably, the healing rate of the 9 µM concentration group was significantly higher than that of the positive control group treated with 12 µM of 5-FU (59.30 ± 1.29%; *p* < 0.01). These findings suggested that **C5** was capable of inhibiting HCT116 cell migration in a concentration-dependent manner.

#### Acute toxicity assay of C5 in vivo

To determine the in vivo safety dose range of compound **C5**, an acute toxicity assay was performed using Kunming mice. Mice were administered a single intraperitoneal injection of **C5** at different doses of 50, 100, 200, 500, and 1000 mg/kg. Toxicological responses and mortality were continuously monitored for 14 consecutive days.

**Fig. 6:**
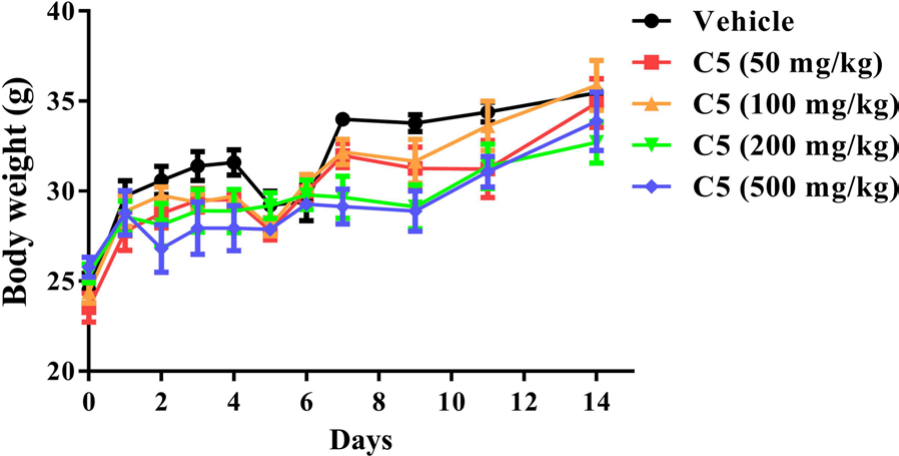
Body weight changes in mice treated with different doses of **C5** during the acute toxicity study. Vehicle (3% DMA/50% PEG400/47% normal saline), **C5** at 50, 100, 200, 500 mg/kg were administered intraperitoneally. Data were expressed as the mean ± SD (n ≥ 4)

As shown in Table 2 and Fig. 6 mice in the 50 and 100 mg/kg groups exhibited no abnormal clinical symptoms or mortality. Obvious toxic reactions and death were observed at doses ≥200 mg/kg. The surviving mice displayed normal feeding, locomotor activity, excretion, and body weight gain during the 14-day observation period, and no delayed toxic manifestations were detected. According to the improved Karber method, the median lethal dose (LD_50_) of compound **C5** in mice was calculated to be 354.81 mg/kg, with a 95% confidence interval of 263.41–477.93 mg/kg. Based on the acute toxicity results, the maximum tolerated dose (MTD) of **C5** in mice was determined to be 100 mg/kg. Following the conventional preclinical dosage design principle for antitumor candidates, 1/10–1/5 of the LD_50_ value was adopted as the safe and effective dose range [33, 34]. Accordingly, three dosage levels of 25, 50, and 75 mg/kg were selected for the in vivo anti-colorectal cancer study, which balanced biosafety evaluation and dose–effect relationship investigation.

**Table 2:** Death of mice in compound **C5** groups

Group	Dose (mg/kg)	Number of deaths		Weight change (%)
		Male	Female	
Vehicle	0	0	0	+ 14.45
C5	50 mg/kg	0	0	+ 14.84
	100 mg/kg	0	0	+ 14.71
	200 mg/kg	2	2	+ 12.87
	500 mg/kg	3	3	+ 13.14
	1000 mg/kg	5	5	0

#### Antitumor activity of C5 in vivo

To evaluate the in vivo antitumor effect of the optimal **C5**, BALB/c nude mice were selected, and HT116 cells were subcutaneously inoculated to establish a human colorectal cancer xenograft mouse model. When the solid tumor volume reached 80–120 mm^3^, the model was considered established, and the mice were randomly divided into five groups. They were intraperitoneally injected with solvent (3% DMA/50% PEG400/47% saline), positive control 5-FU (25 mg/kg), and **C5** at 25, 50, and 75 mg/kg doses, once every two days for a total of 21 days.

**Fig. 7:**
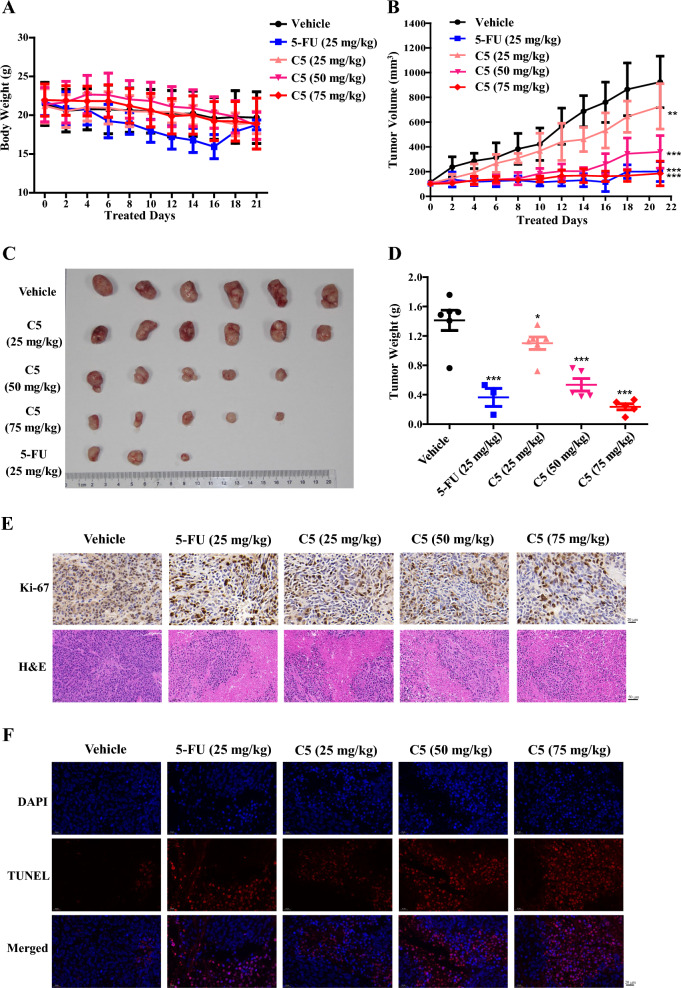

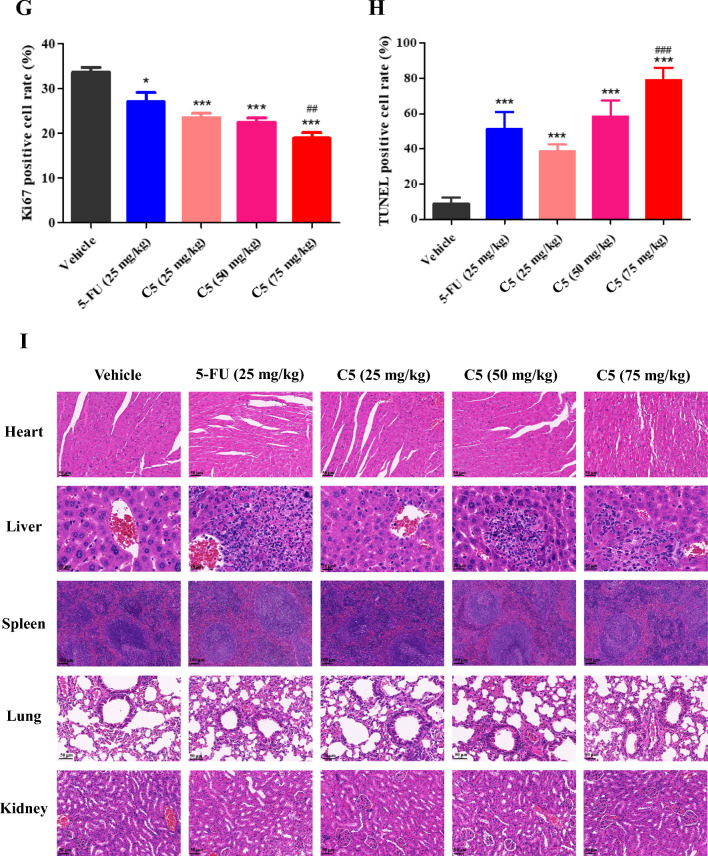
Effect of **C5** on tumor growth of HCT116 xenograft in BALB/c nude mice. Vehicle (3% DMA/50% PEG400/47% normal saline), positive control 5-FU (25 mg/kg), and **C5** at the concentrations of 25 mg/kg, 50 mg/kg, and 75 mg/kg were given respectively, once every two days for 21 consecutive days. **A** The average measured body weight of mice; **B** The tumor volume changes in each group; **C** Images of the isolated tumors derived from mice; **D** The tumor weight and tumor growth inhibition rate; **E** Immunohistochemical staining of Ki-67 and H&E staining in tumor tissues from each group; **F** TUNEL and DAPI fluorescent double staining in tumor tissues from each group; **G** Statistical analysis of Ki-67 positive cell rate in immunohistochemistry; **H** Statistical analysis of TUNEL positive cell rate in TUNEL and DAPI fluorescent double stain; **I** Histopathological lesions were observed by H&E staining on heart (200 ×), liver (400 ×), spleen (100 ×), lung (200 ×) and kidney (200 ×) sections. Data were expressed as the mean ± SD (n = 6). *Compared with the vehicle group, * *p* < 0.05, ** *p* < 0.01, *** *p* < 0.001; ^#^Compared with the 5-FU group, ^#^*p* < 0.05, ^##^*p* < 0.01, ^###^*p* < 0.001

As shown in Fig. 7A, there was no significant difference in the body weight of nude mice between the various dosages of **C5**, the positive control 5-FU group, and the vehicle group during the 21-day treatment period. Figure 7B showed that the tumor volume of the vehicle group gradually increased, reaching 924.50 ± 207.74 mm^3^ after 21 days. In contrast, tumor growth was slower in mice treated with **C5** at 25, 50, and 75 mg/kg, with volumes of 727.17 ± 183.20 mm^3^, 359.20 ± 131.83 mm^3^, and 185.40 ± 99.93 mm^3^, respectively, displaying a dose-dependent effect and significant differences compared to the vehicle group. The high-dose group (75 mg/kg) showed a more significant tumor growth inhibitory effect than the positive control 5-FU group (200.50 ± 80.29 mm^3^). Tumor tissues were collected from each treatment group, and tumor growth inhibition rates were calculated, as shown in Fig. 7C, D. The tumor growth inhibition rates for the low, medium, and high dose groups of** C5** (25, 50 and 75 mg/kg) were 30.76%, 66.28%, and 85.12%, respectively, while the positive control 5-FU group showed a tumor growth inhibition rate of 77.15%. This indicated that the inhibition rate of the medium-dose group of **C5** was similar to that of 5-FU, while the high-dose group outperformed 5-FU, demonstrating a significant inhibitory effect on tumor growth in nude mice. Furthermore, molar dose conversion analysis indicated that the molar dose of **C5** (molecular weight: 478.18) administered at 75 mg/kg was merely 81.59% of that of 5-FU (molecular weight: 130.08) administered at 25 mg/kg. Even at a lower molar dose, **C5** still exhibited a higher tumor growth inhibition rate. From both the mass concentration and molar equivalent perspectives, these data confirmed that **C5** had significantly superior in vivo anti-colorectal cancer efficacy compared with the positive control drug 5-FU.

Immunohistochemical analysis and HE staining were performed on the tumor tissues. The results were shown in Fig. 7E, G. The cells positive for the cell proliferation marker Ki-67 were mostly brown. Compared to the vehicle group, the brown cells were significantly reduced in the tumor sections of the groups treated with various doses of **C5** and the 5-FU group, indicating a significant inhibition of cell proliferation. Moreover, the proliferative inhibitory effect of **C5** was stronger than that of 5-FU. In H&E staining, the tumor cells in the **C5** and 5-FU treatment groups had loose intercellular spaces and reduced trabecular structures, a decreased number of cancer cells with shrunken and deformed nuclei, and visible patchy necrosis with an increased proportion of eosin-stained proteins. Notably, the pathological damage, nuclear pyknosis, and necrotic changes in the high-dose **C5** group were more severe than those in the 5-FU group, further confirming the stronger in vivo antitumor efficacy of compound **C5**. Additionally, TUNEL fluorescence staining was performed on the tumor tissues, and the results were shown in Fig. 7F, H. Compared to the vehicle group, the TUNEL fluorescence staining of the tumor tissue increased significantly with increasing doses of **C5**, indicating that **C5** could induce tumor cell apoptosis and thus exert an antitumor effect.

During the administration period, death occurred in the 5-FU group and the high and medium dose groups of **C5**. Tissue histology analysis was performed on various organs (heart, liver, spleen, lung, and kidney). The results (Fig. 7I) revealed that 5-FU caused liver cell necrosis and severe liver damage in mice, which was consistent with previous literature reports [35]. **C5** also caused liver damage at medium and high doses, but to a lesser extent, a finding that could be mitigated by further structural modification and optimization to minimize the potential hepatotoxicity of **C5**.

#### Inhibitory effect of C5 on PI3K/Akt/mTOR signaling pathways

**Fig. 8:**
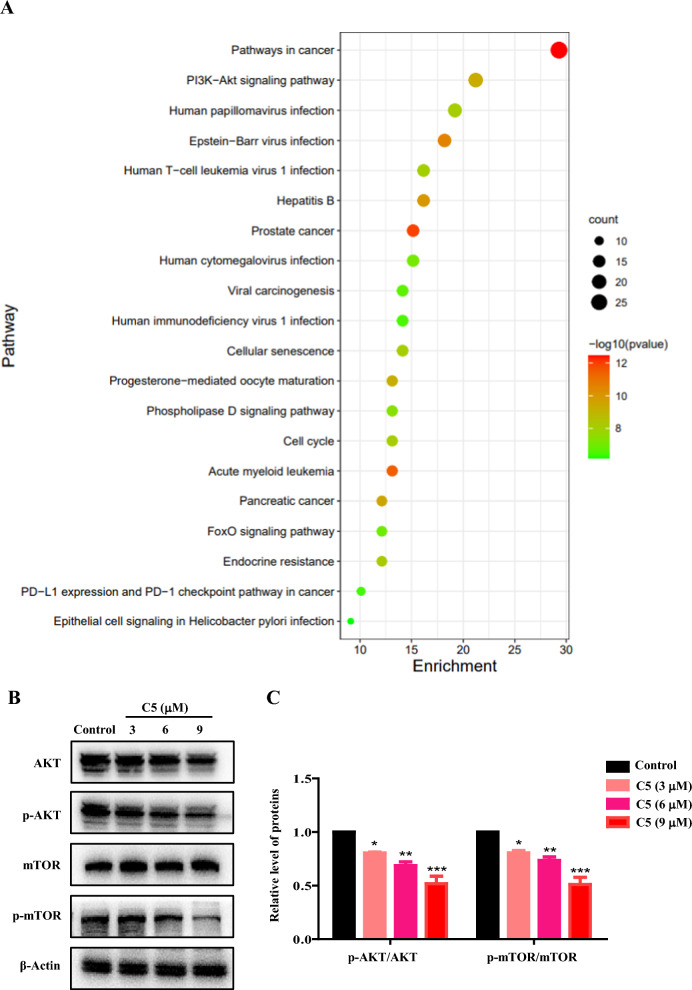
Effect of **C5** on PI3K/Akt signaling pathway. **A** Prediction pathways of **C5** based on KEGG analysis; **B** The changes of proteins expression in HCT116 cells treated with DMSO (control), 5-FU (12 μM) or **C5** (3 μM, 6 μM, and 9 μM) for 48 h by western blotting analysis; **C** Relative levels of proteins were determined (mean ± SD, n = 3), *Compared with the control group, *p < 0.05, **p < 0.01, ***p < 0.001

In this study, we used the SwissTargetPrediction database and KEGG enrichment analysis to predict potential targets and signaling pathways of **C5** [36]. The results showed that **C5** had 102 predicted targets (*P* > 0.1) and affected 89 KEGG pathways (*P* < 0.05). The top 20 pathways with the smallest *P* values were shown in Fig. 8A.

The PI3K/Akt/mTOR pathway was identified as one of the potential pathways affected by **C5**, consistent with previous studies that showed the anticancer effects of THC via regulation of this pathway [37, 38]. Additionally, it has been reported that α, β-unsaturated carbonyl compounds may exert anticancer [21] and anti-inflammatory effects [39] through modulation of the PI3K/Akt/mTOR pathway.

The PI3K/Akt/mTOR pathway is involved in various biological processes of cancer cells and is crucial for tumor development and treatment. Aberrant activation of the PI3K/Akt/mTOR pathway is common in malignant tumors and leads to increased cell proliferation, mitotic stimulation, enhanced metastasis, inhibition of apoptosis, and treatment resistance. Many cytotoxic drugs induce apoptosis in cancer cells by inhibiting the PI3K/Akt/mTOR pathway [40]. Therefore, this pathway has become an attractive target for cancer therapy [41–43].

We performed western blotting to investigate the effects of **C5** on the PI3K/Akt/mTOR pathway. As shown in Fig. 8B, C, compared with the control group, treatment with **C5** significantly decreased the expression of phosphorylation-AKT (p-AKT) and phosphorylation-mTOR (p-mTOR) in HCT116 cells. Moreover, the p-AKT/AKT ratio and p-mTOR/mTOR ratio were significantly decreased in **C5** treatment groups in a concentration-dependent manner. It is known that p-AKT and p-mTOR proteins are mediators of cell proliferation and inhibition of apoptosis. Therefore, **C5** might exert its antitumor effects by inhibiting the PI3K/Akt/mTOR signaling pathway, suppressing cell proliferation, and inducing tumor cell apoptosis.

## Conclusion

In conclusion, we have synthesized a series of THC derivatives bearing α, β-unsaturated carbonyl group to increase their interaction with biological thiols through Michael covalent addition and thereby enhance their antitumor activity. We evaluated the antiproliferative activity of these compounds against HCT116, SW480, SW620, Hela, A549, and HepG2 cell lines. Interestingly, most of the compounds exhibited stronger antiproliferative activity than THC, particularly against colorectal cancer cells. Structure–activity relationship studies also showed that compounds with electron-withdrawing groups exhibited significantly better antiproliferative activity than those with electron-donating groups, and those with electron-withdrawing groups exhibited significantly better anti-colorectal cancer cell proliferation activity, possibly due to increased electrophilicity of the Michael acceptors. Compound **C5**, a para-fluorinated aromatic derivative, exhibited an IC_50_ value of 2.66 ± 0.82 μM against HCT116 cells, which was superior to that of the positive control drug 5-FU.  When the double bond of **C5** was reduced, the antiproliferative activity of the resulting compound **H5** was significantly reduced, indicating that the α, β-unsaturated carbonyl group is an important pharmacophore for the anti-colorectal cancer activity of this series of derivatives. In vivo experiments showed that **C5** significantly inhibited the tumor proliferation and growth of HCT116 in tumor-bearing nude mice, and its effect was comparable to that of the first-line drug 5-FU for anti-CRC activity. Further mechanism studies showed that **C5** inhibited the expression p-AKT and p-mTOR in HCT116 cells, thereby inhibiting the PI3K/AKT/mTOR signaling pathway to induce tumor cell apoptosis, suggesting that **C5** may be a promising lead compound for anti-colorectal cancer drugs. Although **C5** showed significantly lower toxicity compared to 5-FU, it also caused some liver damage in the high-dose group. Therefore, our future efforts will focus on structural optimization of **C5** to improve its ADME/T properties and obtain anti-colorectal cancer candidate drugs with drug-like properties.

## Materials and methods

### Materials and apparatus

In the synthesis of tetrahydrocurcumin derivatives, all reagents and solvents used were from commercial sources without further purification. The flash column chromatography was carried out over silica gel (200–300 mesh) and eluting with ethyl acetate, dichloromethane and petroleum ether (bp 30–60 °C). Thin layer chromatography (TLC) was carried out on glass-backed silica gel plates (Silica Gel 60 Å GF254) and visualized in UV light (λ 254 nm). The structure of the target compound was characterized by nuclear magnetic resonance (^1^H NMR and ^13^C NMR) and high-resolution mass spectrometry (HRMS). NMR spectra were recorded on the Bruker AVANCE instrument (600 MHz) at 25 °C using TMS as the internal standard in CDCl_3_ or DMSO‑d_6_ HRMS was run on an Agilent Q-TOF mass spectrometer.

MTT, Cell cycle, Annexin V-FITC cell apoptosis and BCA protein assay kits were purchased from Beyotime (Shanghai, China). PVDF membranes were purchased from Thermo Scientific. RIPA lysis buffers (K1020), protease inhibitor cocktail I (K1007) and phosphatase inhibitor cocktail I (K1012) were purchased from APExBIO (Houston, USA). ECL luminescent Solution kit was purchased from Affinity (San Antonio, USA). Anti-Bax (50,599-2-Ig-20ul), anti-Bcl-2 (12,789-1-AP-20ul), anti-β-actin (20,536-1-AP), goat anti-rabbit IgG (H + L) (SA00001-2) were purchased from Proteintech (Wuhan, China). Anti-AKT (ab179463), anti-phospho-AKT (ab192623), anti-mTOR (ab134903), anti-phospho- mTOR (ab109268) were purchased from Abcam (Cambridge, UK).

### Experimental animals

Kunming mice aged 4–5 weeks old and BALB/c mice aged 6 weeks were acquired from Laboratory Animal Management Center, Southern Medical University. Before the trial began, the animals were given one week to adapt and had free access to food and water. At the Laboratory Animal Management Center, Southern Medical University, there were places for animals to live. In this work, all animals care and experimental procedures adhered to the Guidelines for the Use of Laboratory animal Care of Southern Medical University. This study was examined and approved by the Animal Ethics Committee of Experimental Animal Ethics Committee of Southern Medical University. The ethical approval number was L2022130.

### General procedure for synthesis of C1-C18

An amount of THC (300 mg, 0.806 mmol, 1 equiv.), indicated benzaldehyde (2 equiv.) as well as toluene (20 mL) were added to a pressure tube. Piperidine (4 μL, 0.04 mmol, 0.05 equiv., in 0.1 mL toluene) and acetic acid (3.7 μL, 0.064 mmol, 0.08 equiv., in 0.1 mL toluene) were added as catalysts. The reaction mixture was stirred in 140 °C. When the reaction done (monitored by TLC), the mixture was washed with water (20 mL, twice) to remove pyridine and acetic acid, and dried over anhydrous Na_2_SO_4_. Next the organic layer was evaporated under vacuum to get raw product. The crude products were purified by silica gel column chromatography using PE/EA (V/V, 1:1) to obtain the target compounds (Scheme 1).

**1,7-Bis(4-hydroxy-3-methoxyphenyl)-4-(4-hydroxybenzylidene)heptane-3,5-dione (C1):** Canary yellow powder, yield 52.43%; ^1^H NMR (500 MHz, DMSO-*d*_6_) *δ*: 10.21 (s, 1H), 8.69 (d, *J* = 5.3 Hz, 2H), 7.59 (s, 1H), 7.19 (d, *J* = 8.7 Hz, 2H), 6.81 (d, *J* = 1.9 Hz, 1H), 6.77–6.72 (m, 2H), 6.71 (d, *J* = 2.0 Hz, 1H), 6.68–6.60 (m, 3H), 6.54 (dd, *J* = 8.0, 2.0 Hz, 1H), 3.73 (s, 3H), 3.70 (s, 3H), 3.05 (t, *J* = 7.6 Hz, 2H), 2.75 (d, *J* = 7.3 Hz, 6H); ^13^C NMR (126 MHz, DMSO-*D*_6_) *δ*: 207.31, 198.68, 160.04, 147.41, 144.69, 144.65, 139.71, 138.53, 132.24, 131.82, 131.40, 123.71, 120.51, 120.46, 115.95, 115.28, 112.72, 112.52, 55.55, 55.49, 45.09, 39.94, 29.49, 28.31; HRMS calculated for C_28_H_28_O_7_ [M + H]^+^ 477.1835, found 477.1906.

**1,7-Bis(4-hydroxy-3-methoxyphenyl)-4-(3-hydroxybenzylidene)heptane-3,5-dione (C2):** Canary yellow powder, yield 72.79%; ^1^H NMR (500 MHz, DMSO-*d*_6_) *δ*: 9.76 (s, 1H), 8.68 (d, *J* = 2.6 Hz, 2H), 7.65 (s, 1H), 7.17 (t, *J* = 7.7 Hz, 1H), 6.86–6.81 (m, 3H), 6.76 (d, *J* = 7.8 Hz, 1H), 6.68–6.64 (m, 2H), 6.62 (dd, *J* = 8.0, 2.7 Hz, 2H), 6.49 (dd, *J* = 8.0, 2.0 Hz, 1H), 3.74 (s, 3H), 3.69 (s, 3H), 3.08 (t, *J* = 7.5 Hz, 2H), 2.77–2.67 (m, 6H); ^13^C NMR (151 MHz, DMSO) *δ*: 207.10, 199.31, 158.06, 147.87, 145.15, 145.09, 141.95, 140.04, 134.80, 132.15, 131.75, 130.46, 121.13, 120.96, 120.71, 118.14, 116.44, 115.74, 115.73, 113.18, 112.76, 56.01, 55.91, 45.69, 40.51, 29.76, 28.89; HRMS calculated for C_28_H_28_O_7_ [M + H]^+^ 477.1835, found 477.1912.

**1,7-Bis(4-hydroxy-3-methoxyphenyl)-4-(4-methoxybenzylidene)heptane-3,5-dione (C3):** Canary yellow powder, yield 31.00%; ^1^H NMR (500 MHz, Chloroform-*d*) *δ*: 7.40 (s, 1H), 7.14 (d, *J* = 8.9 Hz, 2H), 6.84 (d, *J* = 7.9 Hz, 1H), 6.78 (d, *J* = 8.0 Hz, 1H), 6.75 (d, *J* = 8.9 Hz, 2H), 6.71 (d, *J* = 1.9 Hz, 1H), 6.69 (dd, *J* = 7.9, 2.0 Hz, 1H), 6.67 (d, *J* = 2.1 Hz, 1H), 6.61 (dd, *J* = 8.1, 2.0 Hz, 1H), 3.86 (s, 3H), 3.81 (s, 3H), 3.81 (s, 3H), 2.96 (td, *J* = 6.5, 2.3 Hz, 2H), 2.89 (td, *J* = 7.4, 6.9, 3.6 Hz, 4H), 2.79 (t, *J* = 6.9 Hz, 2H); ^13^C NMR (126 MHz, CHLOROFORM-*D*) *δ*: 207.42, 198.02, 161.70, 146.58, 146.45, 144.11, 143.97, 140.02, 139.59, 133.02, 132.71, 131.87, 125.34, 121.32, 120.99, 114.52, 114.36, 111.55, 111.32, 56.02, 55.97, 55.50, 45.94, 40.76, 30.12, 28.98; HRMS calculated for C_29_H_30_O_7_ [M + H]^+^ 491.1992, found 491.2058.

**4-(4-Ethylbenzylidene)-1,7-bis(4-hydroxy-3-methoxyphenyl)heptane-3,5-dione (C4):** Yellow solid, yield 36.91%; ^1^H NMR (400 MHz, Chloroform-*d*) *δ*: 7.45 (s, 1H), 7.12 (q, *J* = 8.9, 7.6 Hz, 4H), 6.84 (d, *J* = 8.0 Hz, 1H), 6.77 (d, *J* = 8.1 Hz, 1H), 6.70 (d, *J* = 13.4 Hz, 2H), 6.64 (d, *J* = 2.3 Hz, 1H), 6.57 (d, *J* = 8.0 Hz, 1H), 5.55 (d, *J* = 8.7 Hz, 2H), 3.87 (s, 3H), 3.81 (s, 3H), 2.95 (d, *J* = 7.2 Hz, 2H), 2.88 (q, *J* = 7.2, 6.7 Hz, 4H), 2.75 (q, *J* = 7.2, 5.8 Hz, 2H), 2.64 (q, *J* = 7.7 Hz, 2H), 1.26–1.20 (m, 3H); ^13^C NMR (101 MHz, CDCl_3_) *δ*: 207.14, 198.09, 147.60, 146.60, 146.46, 144.15, 144.00, 141.35, 139.83, 132.96, 132.64, 130.35, 130.00, 128.61, 121.23, 121.01, 114.53, 114.35, 111.45, 111.34, 56.04, 55.95, 45.98, 40.90, 30.08, 29.06, 28.84, 15.20; HRMS calculated for C_30_H_32_O_6_ [M + H]^+^ 489.2199, found 489.2259.

**4-(4-Fluorobenzylidene)-1,7-bis(4-hydroxy-3-methoxyphenyl)heptane-3,5-dione (C5):** Yellow oil, yield 84.46%; ^1^H NMR (600 MHz, Chloroform-*d*) *δ*: 7.40 (s, 1H), 7.21–7.16 (m, 2H), 6.98–6.92 (m, 2H), 6.84 (d, *J* = 8.0 Hz, 1H), 6.78 (d, *J* = 8.0 Hz, 1H), 6.71 (d, *J* = 1.9 Hz, 1H), 6.69 (dd, *J* = 8.0, 2.0 Hz, 1H), 6.62 (d, *J* = 2.0 Hz, 1H), 6.59 (dd, *J* = 8.1, 2.0 Hz, 1H), 3.87 (s, 3H), 3.81 (s, 3H), 2.95 (ddd, *J* = 7.9, 6.5, 1.7 Hz, 2H), 2.90 (ddd, *J* = 8.6, 6.5, 1.7 Hz, 2H), 2.85 (t, *J* = 7.2 Hz, 2H), 2.76–2.73 (m, 2H); ^13^C NMR (151 MHz, CDCl_3_) *δ*: 206.73, 197.89, 164.77, 163.09, 146.59, 146.47, 144.19, 144.07, 142.09, 138.30, 132.83, 132.43, 131.89, 131.83, 129.20, 129.18, 121.25, 121.00, 116.37, 116.22, 114.53, 114.37, 111.39, 111.31, 77.37, 77.16, 76.95, 56.04, 55.96, 46.05, 40.99, 30.02, 28.97, 0.12; HRMS calculated for C_28_H_27_FO_6_ [M + H]^+^ 479.1792, found 479.1862.

**4-(4-Chlorobenzylidene)-1,7-bis(4-hydroxy-3-methoxyphenyl)heptane-3,5-dione (C6):** Yellow oil, yield 80.68%; ^1^H NMR (600 MHz, Chloroform-*d*) *δ*: 7.39 (s, 1H), 7.24–7.21 (m, 2H), 7.13–7.10 (m, 2H), 6.84 (d, *J* = 8.0 Hz, 1H), 6.80–6.77 (m, 1H), 6.71 (d, *J* = 2.0 Hz, 1H), 6.69 (dd, *J* = 8.0, 2.0 Hz, 1H), 6.59 (d, *J* = 7.4 Hz, 2H), 5.51 (s, 2H), 3.87 (s, 3H), 3.81 (s, 3H), 2.95 (ddd, *J* = 7.9, 6.5, 1.7 Hz, 2H), 2.92–2.88 (m, 2H), 2.85 (t, *J* = 7.2 Hz, 2H), 2.75–2.71 (m, 2H); ^13^C NMR (151 MHz, CDCl_3_) *δ*: 206.53, 197.82, 146.60, 146.49, 144.20, 144.10, 142.70, 138.10, 136.79, 132.78, 132.36, 131.45, 130.89, 129.33, 121.27, 121.00, 114.53, 114.37, 111.33, 111.31, 56.04, 55.95, 46.07, 41.06, 29.99, 28.95, 0.12; HRMS calculated for C_28_H_27_ClO_6_ [M + H]^+^ 495.1496, found 495.1569.

**4-(4-Bromobenzylidene)-1,7-bis(4-hydroxy-3-methoxyphenyl)heptane-3,5-dione (C7):** Yellow oil, yield 67.15%; ^1^H NMR (600 MHz, Chloroform-*d*) *δ*: 7.39 (s, 1H), 7.38–7.36 (m, 2H), 7.06–7.02 (m, 2H), 6.84 (d, *J* = 8.0 Hz, 1H), 6.79 (d, *J* = 8.5 Hz, 1H), 6.70 (d, *J* = 2.0 Hz, 1H), 6.69 (dd, *J* = 8.0, 2.0 Hz, 1H), 6.60–6.57 (m, 2H), 5.51 (s, 2H), 3.87 (s, 3H), 3.81 (s, 3H), 2.96–2.92 (m, 2H), 2.91–2.87 (m, 2H), 2.84 (t, *J* = 7.2 Hz, 2H), 2.72 (t, *J* = 7.0 Hz, 2H); ^13^C NMR (151 MHz, CDCl_3_) *δ*: 206.49, 197.81, 146.59, 146.49, 144.19, 144.10, 142.79, 138.16, 132.76, 132.34, 132.30, 131.88, 131.03, 125.18, 121.28, 121.00, 114.53, 114.37, 111.31, 111.30, 56.04, 55.97, 46.07, 41.07, 29.98, 28.94; HRMS calculated for C_28_H_27_BrO_6_ [M + H]^+^ 539.0991 and 541.0971, found 539.1059 and 541.1041.

**1,7-Bis(4-hydroxy-3-methoxyphenyl)-4-(4-(trifluoromethyl)benzylidene)heptane-3,5-dione (C8):** Yellow oil, yield 93.28%; ^1^H NMR (600 MHz, Chloroform-*d*) *δ*: 7.62 (d, *J* = 7.5 Hz, 1H), 7.56 (s, 1H), 7.44 (s, 1H), 7.37–7.31 (m, 2H), 6.84 (d, *J* = 8.0 Hz, 1H), 6.76 (d, *J* = 8.0 Hz, 1H), 6.71 (d, *J* = 1.9 Hz, 1H), 6.69 (dd, *J* = 8.0, 2.0 Hz, 1H), 6.59 (d, *J* = 2.0 Hz, 1H), 6.55 (dd, *J* = 8.0, 2.0 Hz, 1H), 5.50 (d, *J* = 18.5 Hz, 2H), 3.87 (s, 3H), 3.80 (s, 3H), 2.99–2.95 (m, 2H), 2.90–2.92 (m, 2H), 2.84 (t, *J* = 7.3 Hz, 2H), 2.73 (t, *J* = 7.2 Hz, 2H); ^13^C NMR (151 MHz, CDCl_3_) *δ*: 206.19, 197.83, 146.62, 146.49, 144.25, 144.08, 143.87, 137.50, 133.87, 132.65, 132.29, 132.27, 131.69, 131.48, 129.70, 126.97, 126.95, 126.93, 126.53, 126.50, 124.59, 122.78, 121.09, 121.03, 114.56, 114.40, 111.30, 111.22, 56.04, 55.92, 46.22, 41.11, 29.99, 28.97; HRMS calculated for C_29_H_27_F_3_O_6_ [M + H]^+^ 529.1760, found 529.1830.

**1,7-Bis(4-hydroxy-3-methoxyphenyl)-4-(4-(trifluoromethoxy)benzylidene)heptane-3,5-dione (C9):** Yellow oil, yield 45.79%; ^1^H NMR (400 MHz, Chloroform-*d*) *δ*: 7.40 (s, 1H), 7.21 (d, *J* = 7.4 Hz, 2H), 7.09 (d, *J* = 6.4 Hz, 2H), 6.83 (d, *J* = 8.7 Hz, 1H), 6.77 (d, *J* = 8.7 Hz, 1H), 6.69 (d, *J* = 10.3 Hz, 2H), 6.63 (d, *J* = 3.2 Hz, 1H), 6.58 (d, *J* = 8.3 Hz, 1H), 5.61 (s, 2H), 3.86 (s, 3H), 3.80 (s, 3H), 2.95 (q, *J* = 4.4, 3.8 Hz, 2H), 2.93–2.81 (m, 4H), 2.75 (t, *J* = 5.6 Hz, 2H); ^13^C NMR (101 MHz, CDCl_3_) *δ*: 206.44, 197.90, 150.53, 146.62, 146.49, 144.19, 144.09, 142.85, 137.70, 132.72, 132.32, 131.68, 131.32, 121.23, 120.99, 114.56, 114.40, 111.43, 111.33, 56.00, 55.89, 46.05, 40.99, 29.95, 28.88; HRMS calculated for C_29_H_27_F_3_O_7_ [M + H]^+^ 545.1709, found 545.1771.

**1,7-Bis(4-hydroxy-3-methoxyphenyl)-4-(4-nitrobenzylidene)heptane-3,5-dione (C10):** Orange oil, yield 83.64%; ^1^H NMR (400 MHz, Chloroform-*d*) *δ*: 8.13 (d, *J* = 7.0 Hz, 2H), 7.52 (s, 1H), 7.38 (d, *J* = 7.3 Hz, 2H), 6.89 (dd, *J* = 28.8, 8.1 Hz, 2H), 6.82–6.72 (m, 2H), 6.66 (d, *J* = 6.5 Hz, 2H), 5.79 (s, 2H), 3.96 (s, 3H), 3.85 (s, 3H), 3.13–2.96 (m, 4H), 2.96–2.77 (m, 4H); ^13^C NMR (101 MHz, CDCl_3_) *δ*: 205.57, 197.75, 148.25, 146.63, 146.50, 145.19, 144.20, 144.18, 139.16, 136.39, 132.48, 132.03, 130.10, 123.90, 121.27, 120.94, 114.58, 114.42, 111.36, 56.00, 55.88, 46.08, 41.09, 29.85, 28.62; HRMS calculated for C_28_H_27_NO_8_ [M + H]^+^ 506.1737, found 506.1804.

**1,7-Bis(4-hydroxy-3-methoxyphenyl)-4-(pyridin-4-ylmethylene)heptane-3,5-dione (C11):** Yellow oil, yield 25.21%; ^1^H NMR (400 MHz, Chloroform-*d*) *δ*: 8.50 (s, 2H), 7.30 (s, 1H), 7.01 (s, 2H), 6.83 (d, *J* = 6.9 Hz, 1H), 6.77 (d, *J* = 6.7 Hz, 1H), 6.68 (d, *J* = 4.2 Hz, 2H), 6.62–6.51 (m, 2H), 3.86 (s, 3H), 3.79 (s, 3H), 3.00–2.92 (m, 2H), 2.90 (d, *J* = 5.7 Hz, 2H), 2.82 (d, *J* = 6.3 Hz, 2H), 2.72 (d, *J* = 7.7 Hz, 2H); ^13^C NMR (101 MHz, CDCl_3_) *δ*: 205.40, 197.72, 150.34, 146.71, 146.63, 145.75, 144.33, 144.25, 140.62, 135.98, 132.46, 132.06, 123.13, 121.21, 120.99, 114.67, 114.58, 111.46, 111.38, 56.04, 55.97, 46.14, 41.21, 29.87, 28.80; HRMS calculated for C_27_H_27_NO_6_ [M + H]^+^ 462.1838, found 462.1904.

**1,7-Bis(4-hydroxy-3-methoxyphenyl)-4-(thiophen-3-ylmethylene)heptane-3,5-dione (C12):** Yellow oil, yield 78.23%; ^1^H NMR (500 MHz, Chloroform-*d*) *δ*: 7.38 (s, 1H), 7.33–7.30 (m, 1H), 7.23 (dd, *J* = 5.1, 2.9 Hz, 1H), 6.86 (dd, *J* = 5.1, 1.4 Hz, 1H), 6.84 (d, *J* = 7.9 Hz, 1H), 6.80 (d, *J* = 8.0 Hz, 1H), 6.72–6.67 (m, 3H), 6.64 (dd, *J* = 8.0, 2.0 Hz, 1H), 5.56 (s, 2H), 3.86 (s, 3H), 3.83 (s, 3H), 2.96 (ddd, *J* = 8.0, 6.6, 1.7 Hz, 2H), 2.93–2.91 (m, 2H), 2.91–2.89 (m, 2H), 2.88–2.87 (m, 1H), 2.87–2.84 (m, 1H); ^13^C NMR (126 MHz, CHLOROFORM-*D*) *δ*: 207.04, 198.33, 146.58, 146.49, 144.14, 144.04, 140.48, 134.72, 132.88, 132.75, 132.61, 130.44, 127.76, 127.17, 121.31, 120.97, 114.52, 114.41, 111.54, 111.30, 56.01, 45.94, 40.56, 30.05, 28.93; HRMS (ESI) calculated for C_26_H_26_O_6_S [M + H]^+^ 467.1450, found 467.1525.

**4-(Benzo[b]thiophen-3-ylmethylene)-1,7-bis(4-hydroxy-3-methoxyphenyl)heptane-3,5-dione (C13):** Yellow solid, yield 30.19%; ^1^H NMR (400 MHz, Chloroform-*d*) *δ*: 7.85 (d, *J* = 6.8 Hz, 1H), 7.78 (d, *J* = 6.9 Hz, 1H), 7.72 (s, 1H), 7.44 (t, *J* = 8.7 Hz, 2H), 7.27 (s, 1H), 6.86 (d, *J* = 7.9 Hz, 1H), 6.73 (d, *J* = 11.2 Hz, 3H), 6.58 (d, *J* = 11.1 Hz, 2H), 3.87 (s, 3H), 3.75 (s, 3H), 3.03 (d, *J* = 5.6 Hz, 2H), 3.00–2.91 (m, 2H), 2.90–2.83 (m, 2H), 2.81 (d, *J* = 6.1 Hz, 2H); ^13^C NMR (101 MHz, CDCl_3_) *δ*: 206.89, 197.74, 146.59, 146.45, 144.15, 144.00, 142.46, 139.68, 137.87, 132.75, 132.32, 129.98, 129.95, 128.45, 125.36, 125.02, 122.94, 121.32, 121.24, 120.96, 114.55, 114.41, 111.43, 111.31, 55.95, 55.85, 45.52, 40.90, 30.07, 28.99; HRMS calculated for C_30_H_28_O_6_S [M + H]^+^ 517.1607, found 517.1675.

**4-((1H-pyrrol-3-yl)methylene)-1,7-bis(4-hydroxy-3-methoxyphenyl)heptane-3,5-dione (C14):** Brown solid, yield 52.69%; ^1^H NMR (600 MHz, DMSO-*d*_6_) *δ*: 11.40 (s, 1H), 8.66 (s, 2H), 7.60 (s, 1H), 7.22 (dt, *J* = 3.2, 1.8 Hz, 1H), 6.81 (dd, *J* = 5.1, 2.3 Hz, 2H), 6.75 (d, *J* = 2.0 Hz, 1H), 6.65 (ddd, *J* = 7.2, 6.5, 0.8 Hz, 2H), 6.62 (dd, *J* = 8.1, 1.9 Hz, 1H), 6.56 (dd, *J* = 8.0, 2.0 Hz, 1H), 5.94 (q, *J* = 2.2 Hz, 1H), 3.73 (s, 3H), 3.72 (s, 3H), 2.98 (t, *J* = 7.7 Hz, 2H), 2.84–2.80 (m, 2H), 2.80–2.76 (m, 2H), 2.74 (t, *J* = 7.6 Hz, 2H); ^13^C NMR (151 MHz, DMSO) *δ*: 207.57, 198.22, 147.40, 147.38, 144.63, 144.59, 135.25, 135.12, 131.99, 131.58, 125.64, 120.98, 120.44, 120.31, 117.43, 115.30, 115.26, 112.68, 112.46, 107.75, 55.53, 55.51, 44.86, 38.79, 29.69, 28.45; HRMS calculated for C_26_H_27_NO_6_ [M + H]^+^ 450.1838, found 450.1904.

**4-((1H-indol-3-yl)methylene)-1,7-bis(4-hydroxy-3-methoxyphenyl)heptane-3,5-dione (C15):** Yellow oil, yield 17.41%; ^1^H NMR (400 MHz, Chloroform-*d*) *δ*: 8.85 (s, 1H), 7.77 (s, 1H), 7.66 (d, *J* = 7.4 Hz, 1H), 7.37 (d, *J* = 7.7 Hz, 1H), 7.24 (s, 1H), 6.89 (s, 1H), 6.85 (dd, *J* = 7.8, 2.9 Hz, 1H), 6.82–6.70 (m, 3H), 6.70–6.60 (m, 2H), 5.60 (s, 2H), 3.87 (s, 3H), 3.73 (s, 3H), 3.05 (d, *J* = 6.8 Hz, 2H), 2.94 (d, *J* = 13.5 Hz, 6H); ^13^C NMR (101 MHz, CDCl_3_) *δ*: 208.17, 198.09, 146.64, 146.50, 144.12, 143.73, 137.15, 135.92, 133.21, 133.18, 131.75, 128.00, 127.35, 123.58, 121.61, 121.06, 118.17, 114.57, 114.29, 111.99, 111.88, 111.39, 110.12, 56.03, 55.99, 45.16, 40.25, 30.51, 29.10; HRMS calculated for C_30_H_29_NO_6_ [M + H]^+^500.1995, found 500.2066.

**4-(Furan-2-ylmethylene)-1,7-bis(4-hydroxy-3-methoxyphenyl)heptane-3,5-dione (C16):** Yellow oil, yield 79.84%; ^1^H NMR (600 MHz, Chloroform-*d*) *δ*: 7.34 (d, *J* = 1.7 Hz, 1H), 7.14 (s, 1H), 6.82 (dd, *J* = 11.4, 8.0 Hz, 2H), 6.76 (d, *J* = 1.9 Hz, 1H), 6.71–6.66 (m, 3H), 6.63 (d, *J* = 3.5 Hz, 1H), 6.44 (dd, *J* = 3.5, 1.8 Hz, 1H), 3.86 (s, 3H), 3.85 (s, 3H), 2.99–2.96 (m, 2H), 2.96–2.93 (m, 2H), 2.92–2.89 (m, 2H), 2.89–2.86 (m, 2H); ^13^C NMR (151 MHz, CDCl_3_) *δ*: 205.64, 197.38, 149.02, 146.60, 146.57, 146.45, 144.14, 143.94, 137.83, 133.09, 132.87, 124.77, 121.18, 120.96, 118.40, 114.49, 114.32, 113.08, 111.56, 111.28, 56.02, 45.88, 40.59, 30.02, 28.98; HRMS calculated for C_26_H_26_O_7_ [M + H]^+^ 451.1679, found 451.1749.

**4-(Furan-3-ylmethylene)-1,7-bis(4-hydroxy-3-methoxyphenyl)heptane-3,5-dione (C17):** Orange solid, yield 80.11%; ^1^H NMR (400 MHz, Chloroform-*d*) *δ*: 7.57 (s, 1H), 7.33 (s, 1H), 7.23 (s, 1H), 6.85–6.75 (m, 2H), 6.74–6.62 (m, 4H), 6.15 (s, 1H), 3.83 (d, *J* = 4.3 Hz, 6H), 2.91 (p, *J* = 8.5, 7.3 Hz, 8H); ^13^C NMR (101 MHz, CDCl_3_) *δ*: 206.66, 198.19, 146.59, 146.51, 146.41, 144.66, 144.14, 144.02, 140.61, 132.81, 132.58, 129.90, 121.14, 120.92, 120.19, 114.52, 114.39, 111.43, 111.30, 109.36, 55.98, 45.84, 40.29, 30.00, 28.98; HRMS calculated for C_26_H_26_O_7_ [M + H]^+^ 451.1679, found 451.1751.

**4-(Benzofuran-2-ylmethylene)-1,7-bis(4-hydroxy-3-methoxyphenyl)heptane-3,5-dione (C18):** Yellow powder, yield 22.77%; ^1^H NMR (500 MHz, Chloroform-*d*) *δ*: 7.58 (d, *J* = 7.9 Hz, 1H), 7.40–7.34 (m, 2H), 7.28 (s, 1H), 7.25 (dd, *J* = 7.2, 2.4 Hz, 1H), 6.95 (s, 1H), 6.83 (dd, *J* = 18.4, 8.0 Hz, 2H), 6.73 (d, *J* = 2.0 Hz, 1H), 6.71 (d, *J* = 2.0 Hz, 1H), 6.71–6.70 (m, 1H), 6.69–6.68 (m, 1H), 5.56 (s, 2H), 3.87 (s, 3H), 3.79 (s, 3H), 3.10–3.05 (m, 2H), 3.05–3.00 (m, 2H), 2.98–2.94 (m, 2H), 2.93–2.88 (m, 2H); ^13^C NMR (126 MHz, CHLOROFORM-*D*) *δ*: 205.46, 197.23, 156.23, 150.39, 146.60, 146.54, 144.17, 144.02, 140.22, 132.89, 132.76, 128.06, 127.43, 125.06, 123.94, 122.29, 121.12, 120.97, 114.52, 114.43, 114.39, 111.59, 111.33, 111.29, 56.02, 55.94, 46.25, 40.89, 29.95, 29.26; HRMS calculated for C_30_H_28_O_7_ [M + H]^+^ 501.1835, found 501.1908.

### General procedure for synthesis of H5

360 mg (0.75 mmol) of **C5** and 100 mg 10% Pd/C were added to 2 mL anhydrous ethanol and reacted by stirring at room temperature in hydrogen atmosphere. The reaction mixture was continuously stirred at room temperature for 24 h. When the reaction done (monitored by TLC), the reaction solution was filtered with diatomite and washed with ethanol for many times to get raw product. The crude products were purified by silica gel column chromatography using PE/EA (V/V, 1:2) to obtain the derivatives of **H5** (Scheme 2).

**4-(4-Fluorobenzyl)-1,7-bis(4-hydroxy-3-methoxyphenyl)heptane-3,5-dione** (**H5**)**:** Orange-yellow oil, yield 30.56%; ^1^H NMR (600 MHz, Chloroform-*d*) *δ*: 7.00 (dd, *J* = 8.4, 5.4 Hz, 2H), 6.91 (t, *J* = 8.7 Hz, 2H), 6.79 (d, *J* = 7.9 Hz, 2H), 6.59–6.52 (m, 4H), 5.48 (s, 2H), 3.84 (s, 6H), 3.79 (s, 1H), 3.04 (d, *J* = 7.5 Hz, 2H), 2.67 (dt, *J* = 17.1, 8.9 Hz, 4H), 2.59 (dt, *J* = 24.3, 7.3 Hz, 2H), 2.52–2.45 (m, 2H); ^13^C NMR (151 MHz, CDCl_3_) *δ*: 204.65, 162.58, 146.52, 144.13, 132.45, 130.32, 130.27, 121.01, 120.94, 115.71, 115.57, 114.43, 111.13, 77.37, 77.16, 76.95, 69.53, 56.00, 44.77, 33.51, 29.09; HRMS calculated for C_28_H_29_FO_6_ [M + H]^+^ 481.1982, found 481.2023.

### Biological experiment

#### Cell culture

The cell lines used in our work were human colorectal cancer cell lines (HCT116, SW480, SW620), human cervical cancer cell lines (Hela), human lung adenocarcinoma cancer cell lines (A549), human liver cancer cell lines (HepG2), human intestinal epithelial cell lines (NCM460). All cell lines were obtained from the Cancer Institute, Southern Medical University. Amongst, HCT116 cell line was cultured in McCoýs 5A medium supplemented with 10% fetal bovine serum and 1% penicillin/streptomycin. SW480, Hela, A549 and HepG2 cell lines were cultured in DMEM/high glucose supplemented with 10% fetal bovine serum and 1% penicillin/streptomycin. SW620 and NCM460 cell lines were cultured in RPMI medium 1640 basic supplemented with 10% fetal bovine serum and 1% penicillin/streptomycin. All cell lines were incubated at 37 ℃ and 5% CO_2_ atmosphere.

#### In vitro antiproliferative assay

The in vitro antiproliferative assay of the chemical compounds was measured by the MTT assay. 5 × 10^3^ cells in 100 μL of medium per well were seeded in 96-well plates and incubated for 24 h at 37 °C in 5% CO_2_. Subsequently, the cells were treated with or without the test compounds at different concentrations (1.56–100 μM) diluted with culture medium (a final DMSO concentration ≤ 0.1%) for 72 h. Then, 10 μL of MTT (5 mg/mL) solution was added into each well for another 4 h incubation at 37 °C. Last, the MTT-containing medium was discarded and 150μL of DMSO was added to each well. After the formazan crystals was completely dissolved, the Optical density (OD) of each well was obtained at 490 nm by the microplate reader. The inhibition rates of proliferative were calculated with following equation:

$${\mathrm{Inhibition}}\;{\text{ratio }}\left( {{\% }} \right) = \frac{{{\mathrm{OD}}_{{{\mathrm{control}}}} - {\mathrm{OD}}_{{{\mathrm{treated}}}} }}{{{\mathrm{OD}}_{{{\mathrm{control}}}} }} \times 100$$

The IC_50_ values were calculated by GraphPad Prism 5.0 software. Assays were performed in triplicate wells. Data are presented as the mean ± SD (n = 3).

#### Colorectaly formation assay

For colorectaly formation assay, HCT116 cells (1 × 10^3^ cells/well) were seeded in 6-well plates and cultured for 24 h. Then, the cells were treated with 0.1%DMSO (blank control), 5-FU (12 μM) and different concentrations (3, 6, and 9 μM) of **C5** for 48 h, and subsequently were replaced with complete fresh media which containing 10% FBS for another 7 days’ incubation. Afterwards, the colorectalies were washed thrice with PBS and fixed with 4% paraformaldehyde for 30 min. Next, the colorectalies were stained with 0.1% crystal violet solution for 30 min and then washed for 3–5 times with PBS. Finally, the visible colorectalies were photographed and counted by the software of Image J. Each experiment was repeated at least three times.

#### Cell cycle analysis

Cell cycle analysis kit (Beyotime, China) was used to explore the effect of **C5** on cell cycle. For the cell cycle analysis, HCT116 cells (2 × 10^5^ cells/well) were seeded in 6-well plates and cultured at 37 °C in the 5% CO_2_ incubator for 24 h. Discarding supernatant, and the cells were treated with 0.1% DMSO (blank control), 5-FU (12 μM) and different concentrations (3, 6, and 9 μM) of **C5** for 48 h. The cells were collected and washed in precooled PBS, suspended in ice cold 70% ethanol and stored at 4 °C overnight. After fixing, the cells were washed with PBS at room temperature. Last, the cells were incubated with PI/RNase staining buffer at 37 °C for 30 min in the dark. The DNA content in different groups of cells was assessed by flow cytometry. All experiments were repeated three times.

#### Cell apoptosis analysis

Annexin V/PI Apoptosis Detection Kit (KeyGEN, China) was used to explore the effect of **C5** on the apoptosis of HCT116. For the cell apoptosis analysis, HCT116 cells (2 × 10^5^ cells/well) were seeded in 6-well plates and cultured at 37 °C in the 5% CO_2_ incubator for 24 h. Discarding supernatant, and the cells were treated with 0.1% DMSO (blank control), 5-FU (12 μM) and different concentrations (3, 6, and 9 μM) of **C5** for 48 h. The cells were collected, washed twice with PBS and resuspended in 0.5 mL of binding buffer. Annexin V-FITC (5 μL) and PI (5 μL) were added to the cells, and the mixture were reacted at room temperature for 15 min in the dark. Finally, the apoptotic cells were measured by flow cytometer analysis (BD, USA). All experiments were repeated three times. Annexin-V positive, propidium iodide negative cells were considered early apoptotic and Annexin-V positive, propidium iodide positive cells were considered late apoptotic.

#### Cell migration scratch assay

For wound healing assay, HCT116 cells (5 × 10^5^ cells/well) were seeded in 12-well plates and incubated at 37 °C and 5% CO_2_ atmosphere for 12–24 h. When the cell density is above 90%, the monolayer cells were scraped using 10 μL pipette tips to form scratches. The cells were washed three times with PBS and cultured in serum-free McCoýs 5A medium which containing different concentrations of **C5** and 5-FU (12 μM). The scratches were photographed at 0, 24 and 48 h under the microscope (Lecia) to observe cell migration. The wound areas were analyzed by the software Image J.

#### Western blot analysis

HCT116 cells (5 × 10^5^ cells/well) were seeded in 6 cm dishes and incubated with 24 h. Discarding supernatant, and the cells were treated with 3 mL 0.1% DMSO (blank control), 5-FU (12 μM) and different concentrations (3, 6, and 9 μM) of **C5** for 48 h. After treatment, the cells were collected and lysed in RIPA lysis buffer containing 1% protease and phosphatase inhibitor cocktail I to extract the total proteins. Then the proteins were diluted to 2 mg/mL using BCA protein assay kit. Equal amounts of protein samples were subjected to 10% SDS-PAGE gel for separation, and then transferred to PVDF membrane. After blocking with 5% BSA for 2 h at room temperature, the membranes were incubated with diluted primary antibodies and gently shaken overnight at 4 °C. The membranes were washed with TBST for 30 min (change TBST every 5 min), and then incubated with HRP conjugated secondary antibodies at 4 °C for 2 h. After being washed with TBST buffer again, the protein bands were developed by the super ECL assay kit.

#### Acute toxicity experiment

Following a 6-h fasting period (with water ad libitum), a total of 60 Kunming mice were randomly assigned to 6 groups (10 mice per group, half male and half female) and housed in 10 cages. Each mouse received an intraperitoneal injection at a volume of 0.1 mL per 10 g of body weight. The control group received an intraperitoneal injection of the vehicle (DMA: PEG400: normal saline, V: V: V = 3%: 50%: 47%). The treatment groups were intraperitoneally administered compound 5 at doses of 50, 100, 200, 500, and 1000 mg/kg, respectively. All experimental mice received a single administration and were continuously observed for a period of 14 days. The onset time and characteristics of toxic reactions including mental status, behavior, fur changes, and excretion status were recorded in real time. Body weight variations and the time of death were also documented. In addition, the locomotor activity of the mice was assessed at 0.5, 1, and 2 h post-administration.

#### In vivo tumor growth inhibition assessment

HCT116 cells were cultured and collected during logarithmic phase. BALB/c nude mice (4–5 weeks old) were injected subcutaneously with 5 × 10^6^ cells suspended in 0.1 mL PBS. When the tumor grew to 80-120mm^3^, the nude mice were randomly divided into five groups with 6 mice in each group for HCT116 xenograft model. For vehicle group, mice were i.p. injected with 3% DMA/50% PEG400/47% normal saline. For the positive control group, mice were i.p. injected with 25 mg/kg 5-FU. For the drug groups with different concentrations, mice were i.p. injected with **C5** at 25 mg/kg, 50 mg/kg and 75 mg/kg respectively. The body weight and tumor volume were measured every 2 days. The tumor volume was determined with vernier caliper and the calculated as this formula: A × B^2^/2 (A, long diameter; B, short diameter).

After 21 days, when the mice were sacrificed, the tumors were stripped and weighed. The tumor growth inhibition (TGI) was calculated as TGI = (1−TW_treatment_/TW_vehicle_) × 100%, in which TW_treatment_ is tumor weight of the treatment groups, while TW_vehicle_ is the tumor weight of the vehicle group when the mice were sacrificed. The main tissues, including heart, liver, spleen, lung and kidney, were dissected and washed twice with PBS to remove blood, then fixed in 4% formalin, paraffin embedded and stained with H&E.

#### Immunohistochemistry

The removed tumors of vehicle, 5-FU and **C5** of different concentration treated HCT116 xenograft mice were subject to paraffin-sectioning. Then the sections were stained using H&E staining solution (G1003, ServiceBio, China) or immunostained with Ki-67 antibody (CST, #9449) according to the manufacture’s protocol.

#### Prediction of targets and KEGG pathway enrichment analysis

The potential targets of **C5** were screened out from the SwissTargetPrediction databases and the result with a Probability than 0.1 was selected. The predicted target genes were imported into the DAVID 6.8 (https://david.ncifcrf.gov) database for KEGG pathway enrichment analysis.

#### Statistical analysis

All the experimental data are expressed as mean ± standard deviation (SD, n = 3). Statistical differences between two groups were determined using Student's t-test or Conover's non-parametric test, and statistical analysis was performed using one-way ANOVA using GraphPad Prism. A value of *p* < 0.05 was considered to be statistically significant.

## Supplementary Information

Additional file1 (DOCX 4853 KB)

## Data Availability

Data will be made available on request.
