# Supplementary material for: A novel tetrahydrocurcumin derivative as a potential anti-colorectal cancer agent: studies on its design, synthesis, and in vitro and in vivo biological evaluation
Source: Nat Prod Bioprospect. 2026 Aug 10;16(1):80. doi: 10.1007/s13659-026-00639-7 (PMC13457830; doi:10.1007/s13659-026-00639-7)
Supplement: Supplementary file 1 [file 13659_2026_639_MOESM1_ESM.docx]

**Supplementary Material**

**A novel tetrahydrocurcumin derivative as a potential anti-colorectal agent: Studies on its design, synthesis, and *in vitro* and *in vivo* biological evaluation**

Yixin Chen ^ab, 1^, Ao Duan ^a,1^, Wenzhang Li ^a^, Meitao Duan ^ac^, Jiahui Yu ^a^, Ahmed Mahal ^d^, Yongyan Zhu ^ab*^, Quanhong Zhu ^ab*^

a School of Traditional Chinese Medicine, Southern Medical University, Guangzhou, 510515, China.

b Guangdong Provincial Key Laboratory of Chinese Medicine Pharmaceutics, Guangzhou, 510515, China.

c School of Pharmacy, Xiamen Medical College, Xiamen 361023, China.

d Department of Medical Biochemical Analysis, College of Health Technology, Cihan University Erbil, Erbil, Kurdistan Region, Iraq

^1^ These authors contributed equally to this work.

^*^ Corresponding author: [zqh@smu.edu.cn](mailto:zqh@smu.edu.cn) (Quanhong Zhu.)

yongyanzhu0521@smu.edu.cn (Yongyan Zhu)

**Content**

[**1. Table S1**	1](#_Toc131195029)

[**2.** **HRMS**, **^1^H NMR, and ^13^C NMR spectra for compounds C1-C18, H5**	2](#_Toc131195030)

**1. Table S1**

**Table S1.** The predicted targets of compound **5** by databases of SwissTargetPredition

SwissTargetPredition

ALOX5, BACE1, HTR1A, PDK1, GLO1, EGFR, WEE1, ESRRA, ESRRB, MELK, MDM2, RPS6KB1, AURKA, SPHK2, SPHK1, BMP1, SIGMAR1, TKT, PARP1, DNM1, IKBKG, IKBKB, CHUK, AURKB, CHEK2, ALDH2, SYK, MMP8, TRPM8, HDAC8, PIM1, PIM2, CTSK, ADAM17, ADAM10, CSF1R, EPHX2, CCNE1, CDK2, ADAMTS5, EHMT2, CDK2, CCNA1, CCNA2, NPY5R, PTGS1, TYR, KDM1A, SLC29A1, FCER2, CHEK1, RAF1, AKT2, SLC5A2, BRAF, SLC9A1, ERN1, TNF, AVPR1A, CCND1, CDK4, GCGR, CCNE2, CDK2, CCNE1, JAK1, JAK2, CDC25A, MMP14, HSP90AA1, AVPR2, PDGFRB, CXCR2, TSHR, AGTR1, CDC25C, ALOX5AP, APP, GPR142, CFD, ESR1, ESR2, INSR, FNTA, FNTB, ROCK2, LIMK1, LIMK2, MAPK8, CCNC, CDK8, HCK, PRKCZ, MAPK12, MKNK1, ECE1, CDK8, ALPL, PLAA, HDAC4, NOS1, HSP90AB1, PDE10A

3. **HRMS**, **^1^H NMR, and ^13^C NMR spectra for compounds C1-C18, H5**

**Figure S3.** HRMS spectrum of **C1**

**Figure S4.** ^1^H NMR spectrum of **C1**

**Figure S5.** ^13^C NMR spectrum of **C1**

**Figure S6.** HRMS spectrum of **C2**

**Figure S7.** ^1^H NMR spectrum of **C2**

**Figure S8.** ^13^C NMR spectrum of **C2**

**Figure S9.** HRMS spectrum of **C3**

**Figure S10.** ^1^H NMR spectrum of **C3**

**Figure S11.** ^13^C NMR spectrum of **C3**

**Figure S12.** HRMS spectrum of **C4**

**Figure S13.** ^1^H NMR spectrum of **C4**

**Figure S14.** ^13^C NMR spectrum of **C4**

**Figure S15.** HRMS spectrum of **C5**

**Figure S16.** ^1^H NMR spectrum of **C5**

**Figure S17.** ^13^C NMR spectrum of **C5**

**Figure S18.** HRMS spectrum of **C6**

**Figure S19.** ^1^H NMR spectrum of **C6**

**Figure S20.** ^13^C NMR spectrum of **C6**

**Figure S21.** HRMS spectrum of **C7**

**Figure S22.** ^1^H NMR spectrum of **C7**

**Figure S23.** ^13^C NMR spectrum of **C7**

**Figure S24.** HRMS spectrum of **C8**

**Figure S25.** ^1^H NMR spectrum of **C8**

**Figure S26.** ^13^C NMR spectrum of **C8**

**Figure S27.** HRMS spectrum of **C9**

**Figure S28.** ^1^H NMR spectrum of **C9**

**Figure S29.** ^13^C NMR spectrum of **C9**

**Figure S30.** HRMS spectrum of **C10**

**Figure S31.** ^1^H NMR spectrum of **C10**

**Figure S32.** ^13^C NMR spectrum of **C10**

**Figure S33.** HRMS spectrum of **C11**

**Figure S34.** ^1^H NMR spectrum of **C11**

**Figure S35.** ^13^C NMR spectrum of **C11**

**Figure S36.** HRMS spectrum of **C12**

**Figure S37.** ^1^H NMR spectrum of **C12**

**Figure S38.** ^13^C NMR spectrum of **C12**

**Figure S39.** HRMS spectrum of **C13**

**Figure S40.** ^1^H NMR spectrum of **C13**

**Figure S41.** ^13^C NMR spectrum of **C13**

**Figure S42.** HRMS spectrum of **C14**

**Figure S43.** ^1^H NMR spectrum of **C14**

**Figure S44.** ^13^C NMR spectrum of **C14**

**Figure S45.** HRMS spectrum of **C15**

**Figure S46.** ^1^H NMR spectrum of **C15**

**Figure S47.** ^13^C NMR spectrum of **C15**

**Figure S48.** HRMS spectrum of **C16**

**Figure S49.** ^1^H NMR spectrum of **C16**

**Figure S50.** ^13^C NMR spectrum of **C16**

**Figure S51.** HRMS spectrum of **C17**

**Figure S52.** ^1^H NMR spectrum of **C17**

**Figure S53.** ^13^C NMR spectrum of **C17**

**Figure S54.** HRMS spectrum of **C18**

**Figure S55.** ^1^H NMR spectrum of **C18**

**Figure S56.** ^1^C NMR spectrum of **C18**

**Figure S57.** HRMS spectrum of **H5**

**Figure S58.** ^1^H NMR spectrum of **H5**

**Figure S59.** ^13^C NMR spectrum of **H5**
